# A protocol for research on the use of acupuncture in the management of diabetic peripheral neuropathy in individuals with type 2 diabetes: A systematic review and meta-analysis

**DOI:** 10.1371/journal.pone.0310732

**Published:** 2024-11-14

**Authors:** Xuechun Fan, Jingsi Cao, Guanchi Yan, Yunyun Zhao, Ying Wang, Xiuge Wang, Jia Mi

**Affiliations:** 1 College of Traditional Chinese Medicine, Changchun University of Chinese Medicine, Changchun, China; 2 Department of Endocrinology, The First Affiliated Hospital of Changchun University of Chinese Medicine, Changchun, China; China Medical University, TAIWAN

## Abstract

**Introduction:**

Diabetic peripheral neuropathy (DPN), a prevalent complication among individuals diagnosed with type 2 diabetes, has a significant impact on both the well-being of patients and their financial situation. Acupuncture has been employed for thousands of years within China and is regarded as one of the primary characteristic therapies of traditional Chinese medicine. Research has indicated that acupuncture has the potential to enhance microcirculation, decrease the generation of free radicals, and augment nerve conduction velocity. There had been several meta-analyses of acupuncture on DPN. Nevertheless, there has been inadequate attention given to the assessment of blood glucose control, and scores related to quality of life. Hence, we get additional evidence by enhancing the quantity and quality of studies to draw more distinct findings.

**Methods:**

We will conduct a comprehensive search for reports published from the beginning until June 2023 using various databases including Web of Science, Embase, Cochrane Library, PubMed, AMED, Wanfang database, VIP database, China National Knowledge Infrastructure, and Chinese Biomedical Literature database. Only randomized controlled trials will be considered, with no exclusion of quasi-randomized control trials. Articles in both English and Chinese will be taken into account without any limitations on publication dates. The data will be extracted, managed, and analyzed by two researchers working independently. The primary outcomes will include improvement of symptom scores, change of nerve conduction velocity, and quality of life scores. Additional outcomes will encompass blood glucose levels after fasting and 2 hours after eating, levels of glycosylated hemoglobin, and any adverse events associated with acupuncture. We plan to use the RevMan V.5.4 application and the random-effects model for conducting the meta-analysis. The assessment of potential prejudice can be conducted by Cochrane’s ’risk of bias’ 2 (RoB 2) tool. Registration: PROSPERO (registration number: CRD42023425203).

**Discussion:**

Our goal is to perform a meta-analysis that offers an unbiased approach to treating individuals with type 2 DPN. At the same time, it also provides doctors with more choices in the treatment of diabetes peripheral neuropathy.

## 1 Introduction

The occurrence of type 2 diabetes mellitus (T2DM) is progressively on the rise due to substantial shifts in people’s lifestyle and dietary patterns. Based on the estimates provided by the International Diabetes Federation, approximately 33% of the population is projected to be affected by diabetes by 2050, with nearly 50% of them experiencing neuropathy if glycemic control is not achieved successfully [1, 2]. Diabetic peripheral neuropathy (DPN) has become the prevailing type of neuropathy globally, accounting for the highest occurrence rate [3]. DPN has the potential to cause an array of negative consequences, such as falls and depression [4]. Moreover, DPN additionally raises the likelihood of ulceration, infection, and amputation, thereby adding to the financial burden faced by patients with T2DM (Bewick et al. [Unpublished]) [5]. DPN treatment currently involves managing blood glucose levels, providing relief for pain symptoms, and improving the function of peripheral nerves [6]. Nevertheless, the management of blood glucose levels has limited impact on individuals diagnosed with type 2 DPN [7]. In addition, although many drugs are used to alleviate pain such as antidepressants and anticonvulsants [8], these drugs do not provide significant pain relief and can lead to drug dependence and related serious adverse effects [9, 10].

Hence, it is crucial to discover a efficient remedy with fewer adverse reactions for managing type 2 DPN.

Recently, acupuncture has gained significant popularity and has been extensively employed to treatment for DPN with T2DM, being one of the characteristic therapies in traditional Chinese medicine. Numerous studies have indicated that acupuncture has a clear ability to enhance the nerve conduction speed in individuals diagnosed with T2DM with DPN [11]. In addition, acupuncture has the potential to alleviate pain symptoms in individuals with DPN [12] and enhance microvascular circulation [11]. Hence, acupuncture shows great potential as one of the approach for the clinical management of DPN.

Therefore, our intention is to measure the safety and effectiveness of acupuncture as a therapeutic intervention for individuals experiencing DPN associated to type 2 diabetes. We anticipate that this research will provide valuable insights for the future application of acupuncture in treating individuals with type 2 DPN.

## 2 Materials and methods

### 2.1 Registration of the study

The design of the protocol conforms to the guidelines specified in the Preferred Reporting Items for Systematic Reviews and Meta-analyses Protocols 2015 [13]. This protocol will be performed according to principles outlined in the Cochrane Handbook for Systematic Reviews of Interventions [14]. The study has been registered with the International Prospective Register of Systematic Reviews (PROSPERO). (registration number CRD42023425203).

### 2.2 Criteria for inclusion

#### 2.1.1 Types of study

The research design for this study will incorporate randomized controlled trials (RCTs) that apply acupuncture for the type 2 DPN treatment, while quasi-RCTs will not be specifically excluded. Non-randomized clinical trials, duplicated publications, reviews, conference records, newspapers, meta-analyses, letters, guides, other documents, and studies without full text will be omitted. Publication will be limited to English and Chinese, with no limitations on the date of publication.

#### 2.1.2 Classification of participants

This study will encompass individuals who are adults and have peripheral neuropathy caused by type 2 diabetes. The diagnostic criteria for type 2 diabetes should refer to the 1999 World Health Organization diagnostic criteria for diabetes [15]. There will be no restrictions on the age, gender, ethnic background, or nationality of the participants who are enrolled. Exclusion will occur for studies involving pregnant or individuals with other causes of peripheral neuropathy.

#### 2.1.3 Type of outcomes

The primary outcomes will consist of scores for symptoms (neurological impairment scale [NIS] or similar scales), nerve conduction speed, and scores for life’ quality (Health-Related Quality of Life scale or correlated scores). In addition to acupuncture-related adverse events, the secondary outcomes will encompass fasting blood glucose, blood glucose 2 hours after eating, and glycosylated hemoglobin.

#### 2.1.4 Type of interventions

There will be no restrictions on the selection or positioning of acupuncture points, the technique and strength of acupuncture, the level of stimulation, or the duration and frequency of treatment. We will include randomized controlled trials that meet any of the following intervention comparisons: acupuncture versus placebo or sham treatment, acupuncture combine with other treatment versus other treatment, acupuncture versus no treatment or acupuncture combine with other treatment versus placebo or sham treatment combine with other treatment. ‘Other treatment’ will include psychological treatments, medication, or alternative therapies such as moxibustion etc. The premise is that both the intervention group and the control group receive the same treatment. In addition, we will exclude trials comparing acupuncture with other forms of treatment or only different forms of acupuncture.

### 2.3 Search strategy

The search strategy will be based on the guidelines provided by the Cochrane Handbook for Systematic Reviews of Interventions [14].

### 2.4 Electronic searches

The study will encompass papers published from the inception until June 2023, employing terminology such as acupuncture, DPN, acupoint, and randomized controlled trial. Web of Science, Embase, Cochrane Library, PubMed, AMED, Wanfang database, VIP database, China National Knowledge Infrastructure, and the Chinese Biomedical Literature database will all be queried during the search. Table 1 displays the detailed search method for PubMed.

**Table 1:** Search strategy in PubMed.

Number	Search items
#1	Acupuncture [mh]
#2	Acupuncture therapy [tiab]
#3	Acupuncture, Ear [tiab]
#4	Acupuncture Points [tiab]
#5	Acupuncture Analgesia [tiab]
#6	Acupoint [tiab]
#7	Needling [tiab]
#8	Scalp acupuncture [tiab]
#9	Intradermal needling [tiab]
#10	Auricular acupuncture [tiab]
#11	Ear Acupuncture [tiab]
#12	Electroacupuncture [tiab]
#13	Catgut embedding [tiab]
#14	Manual acupuncture [tiab]
#15	OR/#1—#14
#16	Diabetic neuropathy [mh]
#17	Diabetic Autonomic Neuropathy [tiab]
#18	Painful Diabetic Neuropathy [tiab]
#19	OR/#16—#18
#20	Diabetes mellitus [mh]
#21	Type 2 diabetes mellitus [tiab]
#22	Diabetes mellitus type 2 [tiab]
#23	Diabetes [tiab]
#24	T2D* [tiab]
#25	Diabet* [tiab]
#26	OR/#20—#25
#27	Randomized controlled trial [pt]
#28	Controlled clinical trial [pt]
#29	Randomised [tw]
#30	Randomly [tw]
#31	Trial [tw]
#32	Placebo [tw]
#33	Random allocation [tw]
#34	OR/#27—#33
#35	#15 AND #19 AND #26 AND #34

### 2.5 Other search strategy

We will examine additional ongoing and unpublished studies listed in the WHO International Clinical Trial Registration Platform (https://trialsearch.who.int/), Chinese Clinical Trial Registration (http://www.chictr.org.cn/), and Clinical Trials.gov (https://clinicaltrials.gov/). Additionally, the relevant systematic reviews’ reference lists will be thoroughly examined by manual means. The retrieval of gray literature conducted through the utilization of Google Scholar and opengrey.edu.

### 2.6 Study selection

**Fig 1:**
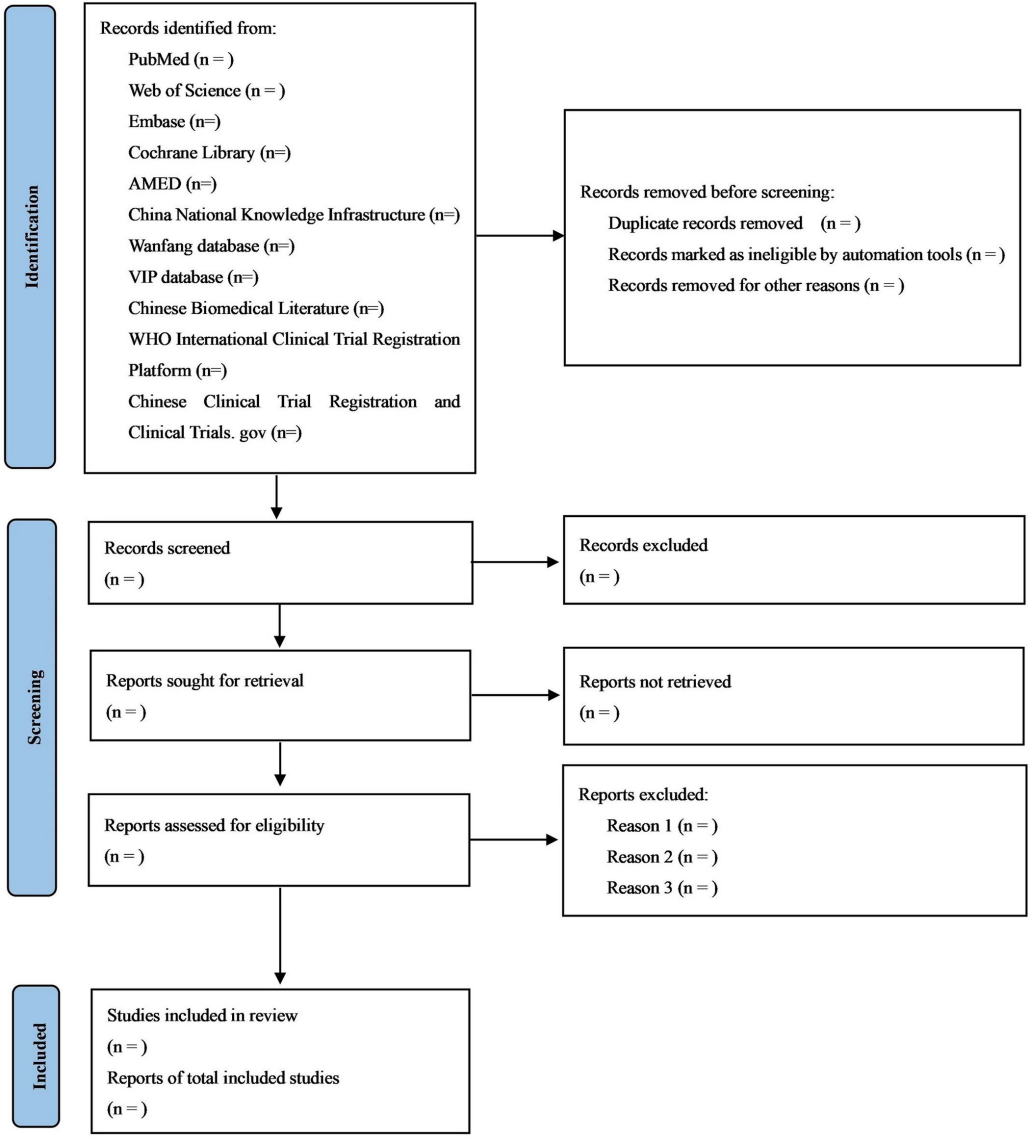
Screening flowchart.

Two researchers will conduct separate evaluations of the studies. The outcomes will be examined and any discrepancies will be discussed. During the course of the research period, any cases of discord will be effectively solved by engaging in discussions or negotiations with a third researcher. Fig 1 displays a flowchart depicting the screening process, which is derived from the PRISMA flow diagram 2020 [16].

### 2.7 Data collection and analysis

After reading the complete text of the chosen papers, two researchers will independently extract the information:

1. The papers contain essential details (the name of primary author, the year of publication, country, language, and race).
2. Key characteristics of the patients (sample size, inclusion criteria, effectiveness criteria, and demographic baseline include age, sex, course of T2DM, duration of type 2 DPN).
3. The details regarding the intervention and control groups, including the duration of intervention, type of intervention, and frequency of intervention.
4. Methodological characteristics (blinding and hidden allocation sequence).
5. The results include primary outcomes (symptom score, nerve conduction velocity, and quality of life scores) and secondary outcomes (2-h postprandial blood glucose, fasting blood glucose, glycosylated hemoglobin, and adverse events).

All conflicts will be settled by participating in discourse or deliberation with a tertiary researcher.

### 2.8 The evaluation of potential biases

The Cochrane Risk of Bias 2 (RoB 2) tool was employed [14], two researchers will individually evaluate the potential for bias. The evaluation and assessment of all the studies will be conducted based on participant blinding, allocation concealment, outcome assessment blinding, result data integrity, selective outcome reporting, along with potential biases. The assessment results will be classified as low, high, or uncertain risk. The classification criteria for bias risk are based on the evaluation results of bias risk in all fields.

#### 2.8.1 Missing data

In case the included studies are missing crucial information, we will reach out to the primary investigator to acquire the necessary details. In the event that the requisite information cannot be acquired, we will exclude these studies.

#### 2.8.2 Data synthesis

RevMan V.5.4 software will be used for meta-analysis. The weighted mean difference of the 95% CI will be used for continuous data and the risk ratios of the 95% CI will be used for dichotomous data. In case there are less than or equal to 3 studies included, we will furnish a descriptive and qualitative overview. Regardless of heterogeneity, a random-effects model was used in all studies.

#### 2.8.3 Analysis of subgroup

Analysis of subgroups will be carried out, taking into account the following factors: acupuncture type, different nerve conduction velocity type, intervention duration, age, sex, course of T2DM, and duration of type 2 DPN.

#### 2.8.4 Sensitivity analysis

To measure the resilience and reliability of the findings, a sensitivity analysis will be conducted. Step-wise rejection method is used for sensitivity analysis.

#### 2.8.5 Meta-regression analysis

Stata will be used for Meta-regression analysis. The process of meta-regression will be carried out in a same manner to linear regression, where the study estimate will serve as the dependent variable and the study characteristics [17] will act as the independent variables.

#### 2.8.6 Evaluation of heterogeneity

To evaluate the heterogeneity of the studies incorporated, we will employ the *χ*^2^ test and *I*^2^ value [14]. When the *P* values in the *χ*^2^ test are greater than 0.05 or the *I*^2^ is less than 50%, it indicates that there is no obvious heterogeneity among the studies. When the value of *P* is less than or equal to 0.05 and the value of *I*^2^ is greater than or equal to 50%, this finding suggests that there is variability or diversity among the studies. To analyze the heterogeneity, we will implement subgroup analysis and meta-regression to analyze the origin of variability. The degree of heterogeneity in studies was evaluated using the *I*^2^ statistic. 0% to 40%: might not be important; 30% to 60%: may represent moderate heterogeneity. 50% to 90%: may represent substantial heterogeneity. 75% to 100%: considerable heterogeneity.

#### 2.8.7 Evaluation of reporting biases

Funnel plots can be utilized to reveal biases of reports when the number of included studies exceeds ten. Funnel plots indicate whether there is publication bias through the symmetry of graphic scatter distribution. When the scatter distribution of the graph is symmetrical, there is no significant publication bias, and vice versa.

### 2.9 Summary of findings

The GRADE will be employed to import data from Review Manager 5.4, in order to generate a table presenting the summary of findings [18]. Two researchers will conduct independent assessments of the evidence’s quality. The downgrading process is based on five domains: study limitations, inconsistency, indirectness of evidence, imprecision and reporting bias. Every result will be categorized into one of four ratings: high, medium, low, or very low.

## 3. Discussion

The incidence of T2DM is progressively escalating as a result of a growing diabetic prevalence. As per the IDF diabetes map for 2021 [19], there has been a significant rise in the global count of adult individuals suffering from diabetes, particularly T2DM, reaching a staggering 537 million [20]. DPN have a significant effect on the life’ quality of patients [21]. Numerous treatment methods have been studied for DPN. Central analgesics are usually used to relieve pain in DPN, but drug dependence is a side effect of this approach. Traditional Chinese Medicine (TCM) is linked to a lower occurrence of adverse reactions compared to conventional medical treatments [22]. Over the past few years, there have been positive reports the acupuncture’ application in the DPN treatment [23–25].

A relevant meta-analysis has been carried out to measure the acupuncture’ effectiveness as a treatment for diabetic peripheral neuropathy [26]. As far as we know, there has not been a specific study conducted to assess the efficacy and safety of acupuncture as a therapeutic intervention for individuals with DPN and T2DM. As the incidence of T2DM is on the rise, healthcare professionals are increasingly interested in comprehending the effectiveness as well as the safety of acupuncture for managing peripheral neuropathy in these individuals.

Hence, we deem it imperative to perform a comprehensive analysis of existing literature through a systematic review and meta-analysis. In our study, we aim assess the efficacy and safety of acupuncture in managing DPN in patients with T2DM. We are confident that clinicians will find the findings of this study valuable. Furthermore, we anticipate that these findings will offer an unbiased approach for treating patients with T2DM-associated DPN. At the same time, it also provides doctors with more choices in the treatment of diabetes peripheral neuropathy.

### 4. Advantages and constraints

This systematic review provides a thorough examination of the efficacy and safety of acupuncture in treating patients with DPN associated with T2DM. Due to the assessment of only RCTs, the inclusion criteria are stringent. To investigate the efficacy of acupuncture, only research studies that utilize acupuncture or a combination of acupuncture and routine treatment (such as stimulating trophic nerves or administering symptomatic therapeutic medications) will be considered. The different types of acupuncture treatments will not have any restrictions, which may result in significant variation among studies.

## Supporting information

S1 Table Search strategy in different databases. (DOCX)
