# Supplementary material for: A protocol for research on the use of acupuncture in the management of diabetic peripheral neuropathy in individuals with type 2 diabetes: A systematic review and meta-analysis
Source: PLoS One. 2024 Nov 14;19(11):e0310732. doi: 10.1371/journal.pone.0310732 (PMC11563441; doi:10.1371/journal.pone.0310732)
Supplement: S1 Table — (DOCX) [file pone.0310732.s001.docx]

Search strategy in different databases

| Search strategy in PubMed | |
| --- | --- |
| Number | Search items |
| #1 | Acupuncture [mh] |
| #2 | (Acupuncture therapy[tiab] OR Acupuncture, Ear[tiab] OR Acupuncture Points[tiab] OR Acupuncture Analgesia[tiab] OR Acupoint[tiab] OR Needling[tiab] OR Scalp acupuncture[tiab] OR Intradermal needling[tiab] OR Auricular acupuncture[tiab] OR Ear Acupuncture[tiab] OR Electroacupuncture[tiab] OR Catgut embedding[tiab] OR Manual acupuncture[tiab]) |
| #3 | OR/#1—#2 |
| #4 | Diabetic neuropathy[mh] |
| #5 | (Neuropathies, Diabetic[tiab] OR Neuropathy, Diabetic[tiab] OR Diabetic Autonomic Neuropathy[tiab] OR Autonomic Neuropathies, Diabetic[tiab] OR Autonomic Neuropathy, Diabetic[tiab] OR Diabetic Autonomic Neuropathies[tiab] OR Neuropathies, Diabetic Autonomic[tiab] OR Neuropathy, Diabetic Autonomic[tiab] OR Diabetic Neuralgia[tiab] OR Diabetic Neuralgias[tiab] OR Neuralgias, Diabetic[tiab] OR Diabetic Neuropathy, Painful[tiab] OR Diabetic Neuropathies, Painful[tiab] OR Neuropathies, Painful Diabetic[tiab] OR Neuropathy, Painful Diabetic[tiab] OR Painful Diabetic Neuropathies[tiab] OR Painful Diabetic Neuropathy[tiab] OR Neuralgia, Diabetic[tiab] OR Symmetric Diabetic Proximal Motor Neuropathy[tiab] OR Asymmetric Diabetic Proximal Motor Neuropathy[tiab] OR Diabetic Asymmetric Polyneuropathy[tiab] OR Asymmetric Polyneuropathies, Diabetic[tiab] OR Asymmetric Polyneuropathy, Diabetic[tiab] OR Diabetic Asymmetric Polyneuropathies[tiab] OR Polyneuropathies, Diabetic Asymmetric[tiab] OR Polyneuropathy, Diabetic Asymmetric[tiab] OR Diabetic Mononeuropathy[tiab] OR Diabetic Mononeuropathies[tiab] OR Mononeuropathies, Diabetic[tiab] OR Mononeuropathy, Diabetic[tiab] OR Diabetic Mononeuropathy Simplex[tiab] OR Diabetic Mononeuropathy Simplices[tiab] OR Mononeuropathy Simplex, Diabetic[tiab] OR Mononeuropathy Simplices, Diabetic[tiab] OR Simplex, Diabetic Mononeuropathy[tiab] OR Simplices, Diabetic Mononeuropathy[tiab] OR Diabetic Amyotrophy[tiab] OR Amyotrophies, Diabetic[tiab] OR Amyotrophy, Diabetic[tiab] OR Diabetic Amyotrophies[tiab] OR Diabetic Polyneuropathy[tiab] OR Diabetic Polyneuropathies[tiab] OR Polyneuropathies, Diabetic[tiab] OR Polyneuropathy, Diabetic[tiab]) |
| #6 | OR/#4—#5 |
| #7 | Type 2 diabetes mellitus [mh] |
| #8 | (Diabetes Mellitus, Noninsulin-Dependent[tiab] OR Diabetes Mellitus, Ketosis-Resistant[tiab] OR Diabetes Mellitus, Ketosis Resistant[tiab] OR Ketosis-Resistant Diabetes Mellitus[tiab] OR Diabetes Mellitus, Non Insulin Dependent[tiab] OR Diabetes Mellitus, Non-Insulin-Dependent[tiab] OR Non-Insulin-Dependent Diabetes Mellitus[tiab] OR Diabetes Mellitus, Stable[tiab] OR Stable Diabetes Mellitus[tiab] OR Diabetes Mellitus, Type II[tiab] OR NIDDM[tiab] OR Diabetes Mellitus, Noninsulin Dependent[tiab] OR Diabetes Mellitus, Maturity-Onset[tiab] OR Diabetes Mellitus, Maturity Onset[tiab] OR Maturity-Onset Diabetes Mellitus[tiab] OR Maturity Onset Diabetes Mellitus[tiab] OR MODY[tiab] OR Diabetes Mellitus, Slow-Onset[tiab] OR Diabetes Mellitus, Slow Onset[tiab] OR Slow-Onset Diabetes Mellitus[tiab] OR Type 2 Diabetes Mellitus[tiab] OR Noninsulin-Dependent Diabetes Mellitus[tiab] OR Noninsulin Dependent Diabetes Mellitus[tiab] OR Maturity-Onset Diabetes[tiab] OR Diabetes, Maturity-Onset[tiab] OR Maturity Onset Diabetes[tiab] OR Type 2 Diabetes[tiab] OR Diabetes, Type 2[tiab] OR Diabetes Mellitus, Adult-Onset[tiab] OR Adult-Onset Diabetes Mellitus[tiab] OR Diabetes Mellitus, Adult Onset [tiab] OR Diabetes Mellitus, Type 2 [tiab] OR T2D* [tiab] OR diabet* [tiab]) |
| #9 | OR/#7—#8 |
| #10 | (randomized controlled trial[pt] OR controlled clinical trial[pt] OR randomised [tw] OR randomly [tw] OR trial [tw] OR placebo[tw] OR random allocation[tw] OR Clinical trial [tiab] OR Clinical study[tiab]) |
| #11 | #3 AND #6 AND #9 AND #10 |
| #12 | "animals"[MeSH Terms] NOT "humans"[MeSH Terms] |
| #13 | #11 NOT #12 |

| Search strategy in Embase | |
| --- | --- |
| Number | Search items |
| #1 | Acupuncture [exp] |
| #2 | Acupuncture therapy [tiab] OR Acupuncture, Ear [tiab] OR Acupuncture Points [tiab] OR Acupuncture Analgesia [tiab] OR Acupoint[tiab] OR Needling[tiab] OR Scalp acupuncture[tiab] OR Intradermal needling[tiab] OR Auricular acupuncture[tiab] OR Ear Acupuncture[tiab] OR Electroacupuncture[tiab] OR Catgut embedding[tiab] OR Manual acupuncture[tiab] |
| #3 | OR/#1—#2 |
| #4 | Diabetic neuropathy[exp] |
| #5 | Neuropathies, Diabetic[tiab] OR Neuropathy, Diabetic[tiab] OR Diabetic Autonomic Neuropathy[tiab] OR Autonomic Neuropathies, Diabetic[tiab] OR Autonomic Neuropathy, Diabetic[tiab] OR Diabetic Autonomic Neuropathies[tiab] OR Neuropathies, Diabetic Autonomic[tiab] OR Neuropathy, Diabetic Autonomic[tiab] OR Diabetic Neuralgia[tiab] OR Diabetic Neuralgias[tiab] OR Neuralgias, Diabetic[tiab] OR Diabetic Neuropathy, Painful[tiab] OR Diabetic Neuropathies, Painful[tiab] OR Neuropathies, Painful Diabetic[tiab] OR Neuropathy, Painful Diabetic[tiab] OR Painful Diabetic Neuropathies[tiab] OR Painful Diabetic Neuropathy[tiab] OR Neuralgia, Diabetic[tiab] OR Symmetric Diabetic Proximal Motor Neuropathy[tiab] OR Asymmetric Diabetic Proximal Motor Neuropathy[tiab] OR Diabetic Asymmetric Polyneuropathy[tiab] OR Asymmetric Polyneuropathies, Diabetic[tiab] OR Asymmetric Polyneuropathy, Diabetic[tiab] OR Diabetic Asymmetric Polyneuropathies[tiab] OR Polyneuropathies, Diabetic Asymmetric[tiab] OR Polyneuropathy, Diabetic Asymmetric[tiab] OR Diabetic Mononeuropathy[tiab] OR Diabetic Mononeuropathies[tiab] OR Mononeuropathies, Diabetic[tiab] OR Mononeuropathy, Diabetic[tiab] OR Diabetic Mononeuropathy Simplex[tiab] OR Diabetic Mononeuropathy Simplices[tiab] OR Mononeuropathy Simplex, Diabetic[tiab] OR Mononeuropathy Simplices, Diabetic[tiab] OR Simplex, Diabetic Mononeuropathy[tiab] OR Simplices, Diabetic Mononeuropathy[tiab] OR Diabetic Amyotrophy[tiab] OR Amyotrophies, Diabetic[tiab] OR Amyotrophy, Diabetic[tiab] OR Diabetic Amyotrophies[tiab] OR Diabetic Polyneuropathy[tiab] OR Diabetic Polyneuropathies[tiab] OR Polyneuropathies, Diabetic[tiab] OR Polyneuropathy, Diabetic[tiab] |
| #6 | OR/#4—#5 |
| #7 | Type 2 diabetes mellitus [exp] |
| #8 | Diabetes Mellitus, Noninsulin-Dependent[tiab] OR Diabetes Mellitus, Ketosis-Resistant[tiab] OR Diabetes Mellitus, Ketosis Resistant[tiab] OR Ketosis-Resistant Diabetes Mellitus[tiab] OR Diabetes Mellitus, Non Insulin Dependent[tiab] OR Diabetes Mellitus, Non-Insulin-Dependent[tiab] OR Non-Insulin-Dependent Diabetes Mellitus[tiab] OR Diabetes Mellitus, Stable[tiab] OR Stable Diabetes Mellitus[tiab] OR Diabetes Mellitus, Type II[tiab] OR NIDDM[tiab] OR Diabetes Mellitus, Noninsulin Dependent[tiab] OR Diabetes Mellitus, Maturity-Onset[tiab] OR Diabetes Mellitus, Maturity Onset[tiab] OR Maturity-Onset Diabetes Mellitus[tiab] OR Maturity Onset Diabetes Mellitus[tiab] OR MODY[tiab] OR Diabetes Mellitus, Slow-Onset[tiab] OR Diabetes Mellitus, Slow Onset[tiab] OR Slow-Onset Diabetes Mellitus[tiab] OR Type 2 Diabetes Mellitus[tiab] OR Noninsulin-Dependent Diabetes Mellitus[tiab] OR Noninsulin Dependent Diabetes Mellitus[tiab] OR Maturity-Onset Diabetes[tiab] OR Diabetes, Maturity-Onset[tiab] OR Maturity Onset Diabetes[tiab] OR Type 2 Diabetes[tiab] OR Diabetes, Type 2[tiab] OR Diabetes Mellitus, Adult-Onset[tiab] OR Adult-Onset Diabetes Mellitus[tiab] OR Diabetes Mellitus, Adult Onset [tiab] OR Diabetes Mellitus, Type 2 [tiab] OR T2D* [tiab] OR diabet* [tiab] |
| #9 | OR/#7—#8 |
| #10 | randomized controlled trial[pt] OR controlled clinical trial[pt] OR randomised [tw] OR randomly [tw] OR trial [tw] OR placebo[tw] OR random allocation[tw] OR Clinical trial [tiab] OR Clinical study[tiab] |
| #11 | #3 AND #6 AND #9 AND #10 |
| #12 | "animals"[exp] NOT "humans"[exp] |
| #13 | #11 NOT #12 |

| Search strategy in Web of Science |
| --- |
| (**TS=** Acupuncture OR Acupuncture therapy OR Acupuncture, Ear OR Acupuncture Points OR Acupuncture Analgesia OR Acupoint OR Needling OR Scalp acupuncture OR Intradermal needling OR Auricular acupuncture OR Ear Acupuncture OR Electroacupuncture OR Catgut embedding OR Manual acupuncture AND TS= Diabetic neuropathy OR Neuropathies, Diabetic OR Neuropathy, Diabetic OR Diabetic Autonomic Neuropathy OR Autonomic Neuropathies, Diabetic OR Autonomic Neuropathy, Diabetic OR Diabetic Autonomic Neuropathies OR Neuropathies, Diabetic Autonomic OR Neuropathy, Diabetic Autonomic OR Diabetic Neuralgia OR Diabetic Neuralgias OR Neuralgias, Diabetic OR Diabetic Neuropathy, Painful OR Diabetic Neuropathies, Painful OR Neuropathies, Painful Diabetic OR Neuropathy, Painful Diabetic OR Painful Diabetic Neuropathies OR Painful Diabetic Neuropathy OR Neuralgia, Diabetic OR Symmetric Diabetic Proximal Motor Neuropathy OR Asymmetric Diabetic Proximal Motor Neuropathy OR Diabetic Asymmetric Polyneuropathy OR Asymmetric Polyneuropathies, Diabetic OR Asymmetric Polyneuropathy, Diabetic OR Diabetic Asymmetric Polyneuropathies OR Polyneuropathies, Diabetic Asymmetric OR Polyneuropathy, Diabetic Asymmetric OR Diabetic Mononeuropathy OR Diabetic Mononeuropathies OR Mononeuropathies, Diabetic OR Mononeuropathy, Diabetic OR Diabetic Mononeuropathy Simplex OR Diabetic Mononeuropathy Simplices OR Mononeuropathy Simplex, Diabetic OR Mononeuropathy Simplices, Diabetic OR Simplex, Diabetic Mononeuropathy OR Simplices, Diabetic Mononeuropathy OR Diabetic Amyotrophy OR Amyotrophies, Diabetic OR Amyotrophy, Diabetic OR Diabetic Amyotrophies OR Diabetic Polyneuropathy OR Diabetic Polyneuropathies OR Polyneuropathies, Diabetic OR Polyneuropathy, Diabetic AND TS= Type 2 diabetes mellitus OR Diabetes Mellitus, Noninsulin-Dependent OR Diabetes Mellitus, Ketosis-Resistant OR Diabetes Mellitus, Ketosis Resistant OR Ketosis-Resistant Diabetes Mellitus OR Diabetes Mellitus, Non Insulin Dependent OR Diabetes Mellitus, Non-Insulin-Dependent OR Non-Insulin-Dependent Diabetes Mellitus OR Diabetes Mellitus, Stable OR Stable Diabetes Mellitus OR Diabetes Mellitus, Type II OR NIDDM OR Diabetes Mellitus, Noninsulin Dependent OR Diabetes Mellitus, Maturity-Onset OR Diabetes Mellitus, Maturity Onset OR Maturity-Onset Diabetes Mellitus OR Maturity Onset Diabetes Mellitus OR MODY OR Diabetes Mellitus, Slow-Onset OR Diabetes Mellitus, Slow Onset OR Slow-Onset Diabetes Mellitus OR Type 2 Diabetes Mellitus OR Noninsulin-Dependent Diabetes Mellitus OR Noninsulin Dependent Diabetes Mellitus OR Maturity-Onset Diabetes OR Diabetes, Maturity-Onset OR Maturity Onset Diabetes OR Type 2 Diabetes OR Diabetes, Type 2 OR Diabetes Mellitus, Adult-Onset OR Adult-Onset Diabetes Mellitus OR Diabetes Mellitus, Adult Onset OR Diabetes Mellitus, Type 2 OR T2D* OR diabet* AND TS= randomized controlled trial OR controlled clinical trial OR randomised OR randomly OR trial OR placebo OR random allocation OR Clinical trial OR Clinical study) **OR**  (**TI=**Acupuncture OR Acupuncture therapy OR Acupuncture, Ear OR Acupuncture Points OR Acupuncture Analgesia OR Acupoint OR Needling OR Scalp acupuncture OR Intradermal needling OR Auricular acupuncture OR Ear Acupuncture OR Electroacupuncture OR Catgut embedding OR Manual acupuncture AND TS= Diabetic neuropathy OR Neuropathies, Diabetic OR Neuropathy, Diabetic OR Diabetic Autonomic Neuropathy OR Autonomic Neuropathies, Diabetic OR Autonomic Neuropathy, Diabetic OR Diabetic Autonomic Neuropathies OR Neuropathies, Diabetic Autonomic OR Neuropathy, Diabetic Autonomic OR Diabetic Neuralgia OR Diabetic Neuralgias OR Neuralgias, Diabetic OR Diabetic Neuropathy, Painful OR Diabetic Neuropathies, Painful OR Neuropathies, Painful Diabetic OR Neuropathy, Painful Diabetic OR Painful Diabetic Neuropathies OR Painful Diabetic Neuropathy OR Neuralgia, Diabetic OR Symmetric Diabetic Proximal Motor Neuropathy OR Asymmetric Diabetic Proximal Motor Neuropathy OR Diabetic Asymmetric Polyneuropathy OR Asymmetric Polyneuropathies, Diabetic OR Asymmetric Polyneuropathy, Diabetic OR Diabetic Asymmetric Polyneuropathies OR Polyneuropathies, Diabetic Asymmetric OR Polyneuropathy, Diabetic Asymmetric OR Diabetic Mononeuropathy OR Diabetic Mononeuropathies OR Mononeuropathies, Diabetic OR Mononeuropathy, Diabetic OR Diabetic Mononeuropathy Simplex OR Diabetic Mononeuropathy Simplices OR Mononeuropathy Simplex, Diabetic OR Mononeuropathy Simplices, Diabetic OR Simplex, Diabetic Mononeuropathy OR Simplices, Diabetic Mononeuropathy OR Diabetic Amyotrophy OR Amyotrophies, Diabetic OR Amyotrophy, Diabetic OR Diabetic Amyotrophies OR Diabetic Polyneuropathy OR Diabetic Polyneuropathies OR Polyneuropathies, Diabetic OR Polyneuropathy, Diabetic AND TS= Type 2 diabetes mellitus OR Diabetes Mellitus, Noninsulin-Dependent OR Diabetes Mellitus, Ketosis-Resistant OR Diabetes Mellitus, Ketosis Resistant OR Ketosis-Resistant Diabetes Mellitus OR Diabetes Mellitus, Non Insulin Dependent OR Diabetes Mellitus, Non-Insulin-Dependent OR Non-Insulin-Dependent Diabetes Mellitus OR Diabetes Mellitus, Stable OR Stable Diabetes Mellitus OR Diabetes Mellitus, Type II OR NIDDM OR Diabetes Mellitus, Noninsulin Dependent OR Diabetes Mellitus, Maturity-Onset OR Diabetes Mellitus, Maturity Onset OR Maturity-Onset Diabetes Mellitus OR Maturity Onset Diabetes Mellitus OR MODY OR Diabetes Mellitus, Slow-Onset OR Diabetes Mellitus, Slow Onset OR Slow-Onset Diabetes Mellitus OR Type 2 Diabetes Mellitus OR Noninsulin-Dependent Diabetes Mellitus OR Noninsulin Dependent Diabetes Mellitus OR Maturity-Onset Diabetes OR Diabetes, Maturity-Onset OR Maturity Onset Diabetes OR Type 2 Diabetes OR Diabetes, Type 2 OR Diabetes Mellitus, Adult-Onset OR Adult-Onset Diabetes Mellitus OR Diabetes Mellitus, Adult Onset OR Diabetes Mellitus, Type 2 OR T2D* OR diabet* AND TS= randomized controlled trial OR controlled clinical trial OR randomised OR randomly OR trial OR placebo OR random allocation OR Clinical trial OR Clinical study) **OR**  **(AB=**Acupuncture OR Acupuncture therapy OR Acupuncture, Ear OR Acupuncture Points OR Acupuncture Analgesia OR Acupoint OR Needling OR Scalp acupuncture OR Intradermal needling OR Auricular acupuncture OR Ear Acupuncture OR Electroacupuncture OR Catgut embedding OR Manual acupuncture AND TS= Diabetic neuropathy OR Neuropathies, Diabetic OR Neuropathy, Diabetic OR Diabetic Autonomic Neuropathy OR Autonomic Neuropathies, Diabetic OR Autonomic Neuropathy, Diabetic OR Diabetic Autonomic Neuropathies OR Neuropathies, Diabetic Autonomic OR Neuropathy, Diabetic Autonomic OR Diabetic Neuralgia OR Diabetic Neuralgias OR Neuralgias, Diabetic OR Diabetic Neuropathy, Painful OR Diabetic Neuropathies, Painful OR Neuropathies, Painful Diabetic OR Neuropathy, Painful Diabetic OR Painful Diabetic Neuropathies OR Painful Diabetic Neuropathy OR Neuralgia, Diabetic OR Symmetric Diabetic Proximal Motor Neuropathy OR Asymmetric Diabetic Proximal Motor Neuropathy OR Diabetic Asymmetric Polyneuropathy OR Asymmetric Polyneuropathies, Diabetic OR Asymmetric Polyneuropathy, Diabetic OR Diabetic Asymmetric Polyneuropathies OR Polyneuropathies, Diabetic Asymmetric OR Polyneuropathy, Diabetic Asymmetric OR Diabetic Mononeuropathy OR Diabetic Mononeuropathies OR Mononeuropathies, Diabetic OR Mononeuropathy, Diabetic OR Diabetic Mononeuropathy Simplex OR Diabetic Mononeuropathy Simplices OR Mononeuropathy Simplex, Diabetic OR Mononeuropathy Simplices, Diabetic OR Simplex, Diabetic Mononeuropathy OR Simplices, Diabetic Mononeuropathy OR Diabetic Amyotrophy OR Amyotrophies, Diabetic OR Amyotrophy, Diabetic OR Diabetic Amyotrophies OR Diabetic Polyneuropathy OR Diabetic Polyneuropathies OR Polyneuropathies, Diabetic OR Polyneuropathy, Diabetic AND TS= Type 2 diabetes mellitus OR Diabetes Mellitus, Noninsulin-Dependent OR Diabetes Mellitus, Ketosis-Resistant OR Diabetes Mellitus, Ketosis Resistant OR Ketosis-Resistant Diabetes Mellitus OR Diabetes Mellitus, Non Insulin Dependent OR Diabetes Mellitus, Non-Insulin-Dependent OR Non-Insulin-Dependent Diabetes Mellitus OR Diabetes Mellitus, Stable OR Stable Diabetes Mellitus OR Diabetes Mellitus, Type II OR NIDDM OR Diabetes Mellitus, Noninsulin Dependent OR Diabetes Mellitus, Maturity-Onset OR Diabetes Mellitus, Maturity Onset OR Maturity-Onset Diabetes Mellitus OR Maturity Onset Diabetes Mellitus OR MODY OR Diabetes Mellitus, Slow-Onset OR Diabetes Mellitus, Slow Onset OR Slow-Onset Diabetes Mellitus OR Type 2 Diabetes Mellitus OR Noninsulin-Dependent Diabetes Mellitus OR Noninsulin Dependent Diabetes Mellitus OR Maturity-Onset Diabetes OR Diabetes, Maturity-Onset OR Maturity Onset Diabetes OR Type 2 Diabetes OR Diabetes, Type 2 OR Diabetes Mellitus, Adult-Onset OR Adult-Onset Diabetes Mellitus OR Diabetes Mellitus, Adult Onset OR Diabetes Mellitus, Type 2 OR T2D* OR diabet* AND TS= randomized controlled trial OR controlled clinical trial OR randomised OR randomly OR trial OR placebo OR random allocation OR Clinical trial OR Clinical study) NOT （**TS=**animals NOT humans OR **TI=** animals NOT humans OR AB**=**animals NOT humans） |

| Search strategy in AMED |
| --- |
| (((((((Acupuncture or Acupuncture therapy or Acupuncture, Ear or Acupuncture Points or Acupuncture Analgesia or Acupoint or Needling or Scalp acupuncture or Intradermal needling or Auricular acupuncture or Ear Acupuncture or Electroacupuncture or Catgut embedding or Manual acupuncture).af. and Diabetic neuropathy.af.) or Neuropathies, Diabetic.af.or Neuropathy, Diabetic.af.or Diabetic Autonomic Neuropathy.af. or Autonomic Neuropathies, Diabetic.af.or Autonomic Neuropathy, Diabetic.af.or Diabetic Autonomic Neuropathies.af.or Neuropathies, Diabetic Autonomic.af.or Neuropathy, Diabetic Autonomic.af.or Diabetic Neuralgia.af. or Diabetic Neuralgias.af.or Neuralgias, Diabetic.af.or Diabetic Neuropathy, Painful.af.or Diabetic Neuropathies, Painful.af.or Neuropathies, Painful Diabetic.af.or Neuropathy, Painful Diabetic.af.or Painful Diabetic Neuropathies.af.or Painful Diabetic Neuropathy.af.or Neuralgia, Diabetic.af.or Symmetric Diabetic Proximal Motor Neuropathy.af.or Asymmetric Diabetic Proximal Motor Neuropathy.af.or Diabetic Asymmetric Polyneuropathy.af.or Asymmetric Polyneuropathies, Diabetic.af.or Asymmetric Polyneuropathy, Diabetic.af.or Diabetic Asymmetric Polyneuropathies.af.or Polyneuropathies, Diabetic Asymmetric.af.or Polyneuropathy, Diabetic Asymmetric.af.or Diabetic Mononeuropathy.af.or Diabetic Mononeuropathies.af.or Mononeuropathies, Diabetic.af.or Mononeuropathy, Diabetic.af.or Diabetic Mononeuropathy Simplex.af.or Diabetic Mononeuropathy Simplices.af.or Mononeuropathy Simplex, Diabetic.af.or Mononeuropathy Simplices, Diabetic.af.or Simplex, Diabetic Mononeuropathy.af.or Simplices, Diabetic Mononeuropathy.af.or Diabetic Amyotrophy.af.or Amyotrophies, Diabetic.af.or Amyotrophy, Diabetic.af.or Diabetic Amyotrophies.af.or Diabetic Polyneuropathy.af.or Diabetic Polyneuropathies.af.or Polyneuropathies, Diabetic.af. or Polyneuropathy, Diabetic.af.)and Type 2 diabetes mellitus.af.)or Diabetes Mellitus, Noninsulin-Dependent.af.or Diabetes Mellitus, Ketosis-Resistant.af.or Diabetes Mellitus, Ketosis Resistant.af.or Ketosis-Resistant Diabetes Mellitus.af.or Diabetes Mellitus, Non Insulin Dependent.af.or Diabetes Mellitus, Non-Insulin-Dependent.af.or Non-Insulin-Dependent Diabetes Mellitus.af.or Diabetes Mellitus, Stable .af.or Stable Diabetes Mellitus.af. or Diabetes Mellitus, Type II.af.or NIDDM.af. or Diabetes Mellitus, Noninsulin Dependent.af.or Diabetes Mellitus, Maturity-Onset.af.or Diabetes Mellitus, Maturity Onset.af.or Maturity-Onset Diabetes Mellitus.af.or Maturity Onset Diabetes Mellitus.af. or MODY.af.or Diabetes Mellitus, Slow-Onset.af. or Diabetes Mellitus, Slow Onset.af.or Slow-Onset Diabetes Mellitus.af. or Type 2 Diabetes Mellitus.af.or Noninsulin-Dependent Diabetes Mellitus.af.or Noninsulin Dependent Diabetes Mellitus.af.or Maturity-Onset Diabetes.af.or Diabetes, Maturity-Onset.af.or Maturity Onset Diabetes.af.or Type 2 Diabetes.af.or Diabetes, Type 2.af. or Diabetes Mellitus, Adult-Onset.af. or Adult-Onset Diabetes Mellitus.af.or Diabetes Mellitus, Adult Onset.af. or Diabetes Mellitus, Type 2.af. or T2D*.af.or diabet*.af.) and randomized controlled trial.af.) or controlled clinical trial.af.or randomised.af. or randomly.af. or trial.af. or placebo.af. or random allocation.af. or Clinical trial.af. or Clinical study.af.)  **or**  (((((((Acupuncture or Acupuncture therapy or Acupuncture, Ear or Acupuncture Points or Acupuncture Analgesia or Acupoint or Needling or Scalp acupuncture or Intradermal needling or Auricular acupuncture or Ear Acupuncture or Electroacupuncture or Catgut embedding or Manual acupuncture) .ab. and Diabetic neuropathy.ab.) or Neuropathies, Diabetic.ab.or Neuropathy, Diabetic.ab.or Diabetic Autonomic Neuropathy.ab. or Autonomic Neuropathies, Diabetic.ab.or Autonomic Neuropathy, Diabetic.ab.or Diabetic Autonomic Neuropathies.ab.or Neuropathies, Diabetic Autonomic.ab.or Neuropathy, Diabetic Autonomic.ab.or Diabetic Neuralgia.ab.or Diabetic Neuralgias.ab.or Neuralgias, Diabetic.ab.or Diabetic Neuropathy, Painful.ab.or Diabetic Neuropathies, Painful.ab.or Neuropathies, Painful Diabetic.ab.or Neuropathy, Painful Diabetic.ab.or Painful Diabetic Neuropathies.ab.or Painful Diabetic Neuropathy.ab.or Neuralgia, Diabetic.ab.or Symmetric Diabetic Proximal Motor Neuropathy.ab.or Asymmetric Diabetic Proximal Motor Neuropathy.ab.or Diabetic Asymmetric Polyneuropathy.ab.or Asymmetric Polyneuropathies, Diabetic.ab.or Asymmetric Polyneuropathy, Diabetic.ab.or Diabetic Asymmetric Polyneuropathies.ab.or Polyneuropathies, Diabetic Asymmetric.ab.or Polyneuropathy, Diabetic Asymmetric.ab.or Diabetic Mononeuropathy.ab.or Diabetic Mononeuropathies.ab.or Mononeuropathies, Diabetic.ab.or Mononeuropathy, Diabetic.ab.or Diabetic Mononeuropathy Simplex.ab.or Diabetic Mononeuropathy Simplices.ab.or Mononeuropathy Simplex, Diabetic.ab.or Mononeuropathy Simplices, Diabetic.ab.or Simplex, Diabetic Mononeuropathy.ab.or Simplices, Diabetic Mononeuropathy.ab.or Diabetic Amyotrophy.ab.or Amyotrophies, Diabetic.ab. or Amyotrophy, Diabetic.ab.or Diabetic Amyotrophies.ab. or Diabetic Polyneuropathy.ab. or Diabetic Polyneuropathies.ab. or Polyneuropathies, Diabetic.ab. or Polyneuropathy, Diabetic.ab.)and Type 2 diabetes mellitus.ab.)or Diabetes Mellitus, Noninsulin-Dependent.ab. or Diabetes Mellitus, Ketosis-Resistant.ab. or Diabetes Mellitus, Ketosis Resistant.ab. or Ketosis-Resistant Diabetes Mellitus.ab. or Diabetes Mellitus, Non Insulin Dependent.ab. or Diabetes Mellitus, Non-Insulin-Dependent.ab. or Non-Insulin-Dependent Diabetes Mellitus.ab. or Diabetes Mellitus, Stable .ab. or Stable Diabetes Mellitus.ab. or Diabetes Mellitus, Type II.ab. or NIDDM.ab. or Diabetes Mellitus, Noninsulin Dependent.ab. or Diabetes Mellitus, Maturity-Onset.ab. or Diabetes Mellitus, Maturity Onset.ab. or Maturity-Onset Diabetes Mellitus.ab. or Maturity Onset Diabetes Mellitus.ab. or MODY.ab. or Diabetes Mellitus, Slow-Onset.ab. or Diabetes Mellitus, Slow Onset.ab. or Slow-Onset Diabetes Mellitus.ab. or Type 2 Diabetes Mellitus.ab.or Noninsulin-Dependent Diabetes Mellitus.ab.or Noninsulin Dependent Diabetes Mellitus.ab. or Maturity-Onset Diabetes.ab. or Diabetes, Maturity-Onset.ab. or Maturity Onset Diabetes.ab. or Type 2 Diabetes.ab. or Diabetes, Type 2.ab. or Diabetes Mellitus, Adult-Onset.ab. or Adult-Onset Diabetes Mellitus.ab.or Diabetes Mellitus, Adult Onset.ab. or Diabetes Mellitus, Type 2.ab. or T2D*.ab. or diabet*.ab.) and randomized controlled trial.ab.) or controlled clinical trial.ab. or randomised.ab. or randomly.ab. or trial.ab. or placebo.ab. or random allocation.ab. or Clinical trial.ab. or Clinical study.ab.)  **or**  (((((((Acupuncture or Acupuncture therapy or Acupuncture, Ear or Acupuncture Points or Acupuncture Analgesia or Acupoint or Needling or Scalp acupuncture or Intradermal needling or Auricular acupuncture or Ear Acupuncture or Electroacupuncture or Catgut embedding or Manual acupuncture) .at. and Diabetic neuropathy. at.) or Neuropathies, Diabetic.at.or Neuropathy, Diabetic.at.or Diabetic Autonomic Neuropathy.at. or Autonomic Neuropathies, Diabetic.at.or Autonomic Neuropathy, Diabetic.at.or Diabetic Autonomic Neuropathies.at.or Neuropathies, Diabetic Autonomic.at.or Neuropathy, Diabetic Autonomic.at. or Diabetic Neuralgia.at. or Diabetic Neuralgias.at. or Neuralgias, Diabetic.at. or Diabetic Neuropathy, Painful.at. or Diabetic Neuropathies, Painful.at. or Neuropathies, Painful Diabetic.at. or Neuropathy, Painful Diabetic.at. or Painful Diabetic Neuropathies.at. or Painful Diabetic Neuropathy.at. or Neuralgia, Diabetic.at. or Symmetric Diabetic Proximal Motor Neuropathy.at. or Asymmetric Diabetic Proximal Motor Neuropathy.at. or Diabetic Asymmetric Polyneuropathy.at. or Asymmetric Polyneuropathies, Diabetic.at. or Asymmetric Polyneuropathy, Diabetic.at. or Diabetic Asymmetric Polyneuropathies.at. or Polyneuropathies, Diabetic Asymmetric.at. or Polyneuropathy, Diabetic Asymmetric.at. or Diabetic Mononeuropathy.at. or Diabetic Mononeuropathies.at. or Mononeuropathies, Diabetic.at. or Mononeuropathy, Diabetic.at. or Diabetic Mononeuropathy Simplex.at. or Diabetic Mononeuropathy Simplices.at. or Mononeuropathy Simplex, Diabetic.at. or Mononeuropathy Simplices, Diabetic.at. or Simplex, Diabetic Mononeuropathy.at. or Simplices, Diabetic Mononeuropathy.at. or Diabetic Amyotrophy.at. or Amyotrophies, Diabetic.at. or Amyotrophy, Diabetic.at. or Diabetic Amyotrophies.at. or Diabetic Polyneuropathy.at. or Diabetic Polyneuropathies.at. or Polyneuropathies, Diabetic.at. or Polyneuropathy, Diabetic.at.)and Type 2 diabetes mellitus.at.)or Diabetes Mellitus, Noninsulin-Dependent.at. or Diabetes Mellitus, Ketosis-Resistant.at. or Diabetes Mellitus, Ketosis Resistant.at. or Ketosis-Resistant Diabetes Mellitus.at. or Diabetes Mellitus, Non Insulin Dependent.at. or Diabetes Mellitus, Non-Insulin-Dependent.at. or Non-Insulin-Dependent Diabetes Mellitus.at. or Diabetes Mellitus, Stable .at. or Stable Diabetes Mellitus.at. or Diabetes Mellitus, Type II.at. or NIDDM.at. or Diabetes Mellitus, Noninsulin Dependent.at. or Diabetes Mellitus, Maturity-Onset.at. or Diabetes Mellitus, Maturity Onset.at. or Maturity-Onset Diabetes Mellitus.at. or Maturity Onset Diabetes Mellitus.at. or MODY.at. or Diabetes Mellitus, Slow-Onset.at. or Diabetes Mellitus, Slow Onset.at. or Slow-Onset Diabetes Mellitus.at. or Type 2 Diabetes Mellitus.at. or Noninsulin-Dependent Diabetes Mellitus.at. or Noninsulin Dependent Diabetes Mellitus.at. or Maturity-Onset Diabetes.at. or Diabetes, Maturity-Onset.at. or Maturity Onset Diabetes.at. or Type 2 Diabetes.at. or Diabetes, Type 2.at. or Diabetes Mellitus, Adult-Onset.at. or Adult-Onset Diabetes Mellitus.at.or Diabetes Mellitus, Adult Onset.at. or Diabetes Mellitus, Type 2.at. or T2D*.at. or diabet*.at.) and randomized controlled trial.at.) or controlled clinical trial.at. or randomised.at. or randomly.at. or trial.at. or placebo.at. or random allocation.at. or Clinical trial.at. or Clinical study.at.) not（((((animals.af.） not humans.af.) or animals.ab.) not humans.ab.) or animals.at.) not humans.at. |

| Search strategy in Cochrane Library | |
| --- | --- |
| Number | Search items |
| #1 | MeSH descriptor : [Acupuncture] explode all trees |
| #2 | (Acupuncture therapy OR Acupuncture, Ear OR Acupuncture Points OR Acupuncture Analgesia OR Acupoint OR Needling OR Scalp acupuncture OR Intradermal needling OR Auricular acupuncture OR Ear Acupuncture OR Electroacupuncture OR Catgut embedding OR Manual acupuncture): ti,ab, kw |
| #3 | OR/#1—#2 |
| #4 | MeSH descriptor : [Diabetic neuropathy] explode all trees |
| #5 | (Neuropathies, Diabetic OR Neuropathy, Diabetic OR Diabetic Autonomic Neuropathy OR Autonomic Neuropathies, Diabetic OR Autonomic Neuropathy, Diabetic OR Diabetic Autonomic Neuropathies OR Neuropathies, Diabetic Autonomic OR Neuropathy, Diabetic Autonomic OR Diabetic Neuralgia OR Diabetic Neuralgias OR Neuralgias, Diabetic OR Diabetic Neuropathy, Painful OR Diabetic Neuropathies, Painful OR Neuropathies, Painful Diabetic OR Neuropathy, Painful Diabetic OR Painful Diabetic Neuropathies OR Painful Diabetic Neuropathy OR Neuralgia, Diabetic OR Symmetric Diabetic Proximal Motor Neuropathy OR Asymmetric Diabetic Proximal Motor Neuropathy OR Diabetic Asymmetric Polyneuropathy OR Asymmetric Polyneuropathies, Diabetic OR Asymmetric Polyneuropathy, Diabetic OR Diabetic Asymmetric Polyneuropathies OR Polyneuropathies, Diabetic Asymmetric OR Polyneuropathy, Diabetic Asymmetric OR Diabetic Mononeuropathy OR Diabetic Mononeuropathies OR Mononeuropathies, Diabetic OR Mononeuropathy, Diabetic OR Diabetic Mononeuropathy Simplex OR Diabetic Mononeuropathy Simplices OR Mononeuropathy Simplex, Diabetic OR Mononeuropathy Simplices, Diabetic OR Simplex, Diabetic Mononeuropathy OR Simplices, Diabetic Mononeuropathy OR Diabetic Amyotrophy OR Amyotrophies, Diabetic OR Amyotrophy, Diabetic OR Diabetic Amyotrophies OR Diabetic Polyneuropathy OR Diabetic Polyneuropathies OR Polyneuropathies, Diabetic OR Polyneuropathy, Diabetic): ti,ab, kw |
| #6 | OR/#4—#5 |
| #7 | MeSH descriptor : [Type 2 diabetes mellitus] explode all trees |
| #8 | (Diabetes Mellitus, Noninsulin-Dependent OR Diabetes Mellitus, Ketosis-Resistant OR Diabetes Mellitus, Ketosis Resistant OR Ketosis-Resistant Diabetes Mellitus OR Diabetes Mellitus, Non Insulin Dependent OR Diabetes Mellitus, Non-Insulin-Dependent OR Non-Insulin-Dependent Diabetes Mellitus OR Diabetes Mellitus, Stable OR Stable Diabetes Mellitus OR Diabetes Mellitus, Type II OR NIDDM OR Diabetes Mellitus, Noninsulin Dependent OR Diabetes Mellitus, Maturity-Onset OR Diabetes Mellitus, Maturity Onset OR Maturity-Onset Diabetes Mellitus OR Maturity Onset Diabetes Mellitus OR MODY OR Diabetes Mellitus, Slow-Onset OR Diabetes Mellitus, Slow Onset OR Slow-Onset Diabetes Mellitus OR Type 2 Diabetes Mellitus OR Noninsulin-Dependent Diabetes Mellitus OR Noninsulin Dependent Diabetes Mellitus OR Maturity-Onset Diabetes OR Diabetes, Maturity-Onset OR Maturity Onset Diabetes OR Type 2 Diabetes OR Diabetes, Type 2 OR Diabetes Mellitus, Adult-Onset OR Adult-Onset Diabetes Mellitus OR Diabetes Mellitus, Adult Onset OR Diabetes Mellitus, Type 2 OR T2D* OR diabet*): ti,ab, kw |
| #9 | OR/#7—#8 |
| #10 | (randomized controlled trial OR controlled clinical trial OR randomised OR randomly OR trial OR placebo OR random allocation OR Clinical trial OR Clinical study): ti,ab, kw |
| #11 | #3 AND #6 AND #9 AND #10 |
| #12 | MeSH descriptor :"animals" explode all trees NOT MeSH descriptor :"humans" explode all trees |
| #13 | #11 NOT #12 |

| Search strategy in CNKI |
| --- |
| (**SU=**针刺+针灸+电针+温针+温针灸+体针+头针+耳针+穴位 AND **SU=** 2型糖尿病+非依赖胰岛素型糖尿病+成人发病型糖尿病+消渴+消渴病 AND **SU=** 糖尿病性周围神经病+糖尿病周围神经病+糖尿病周围神经病变 AND **SU=**2型糖尿病性周围神经病+2型糖尿病性周围神经病变+消渴病痹症+消渴痹症+消渴病痹证+消渴痹证+周痹 AND **SU=**随机对照试验+随机+对照+试验+临床疗效观察+疗效+临床+治疗+影响)**OR**  (KY**=**针刺+针灸+电针+温针+温针灸+体针+头针+耳针+穴位 AND **KY=** 2型糖尿病+非依赖胰岛素型糖尿病+成人发病型糖尿病+消渴+消渴病 AND **KY=** 糖尿病性周围神经病+糖尿病周围神经病+糖尿病周围神经病变AND KY**=**2型糖尿病性周围神经病+2型糖尿病性周围神经病变+消渴病痹症+消渴痹症+消渴病痹证+消渴痹证+周痹AND KY**=**随机对照试验+随机+对照+试验+临床疗效观察+疗效+临床+治疗+影响) **OR**  (TI**=**针刺+针灸+电针+温针+温针灸+体针+头针+耳针+穴位 AND TI**=** 2型糖尿病+非依赖胰岛素型糖尿病+成人发病型糖尿病+消渴+消渴病 AND **TI=** 糖尿病性周围神经病+糖尿病周围神经病+糖尿病周围神经病变AND TI**=**2型糖尿病性周围神经病+2型糖尿病性周围神经病变+消渴病痹症+消渴痹症+消渴病痹证+消渴痹证+周痹AND TI**=**随机对照试验+随机+对照+试验+临床疗效+临床疗效观察+疗效+临床+治疗+影响) NOT (**SU=**动物NOT人类+人OR KY**=**动物NOT人类+人OR TI**=**动物NOT人类+人) |

| Search strategy in Wanfang |
| --- |
| 主题：（（“针刺”OR“针灸”OR“电针”OR “温针”OR “温针灸”OR “体针”OR “头针”OR “耳针”OR “穴位”）AND （“2型糖尿病”OR “非依赖胰岛素型糖尿病”OR “成人发病型糖尿病”OR “消渴”OR “消渴病”） AND （“糖尿病性周围神经病”OR “糖尿病周围神经病”OR “糖尿病周围神经病变”）AND（“2型糖尿病性周围神经病”OR “2型糖尿病性周围神经病变”OR “消渴病痹症”OR “消渴痹症”OR “消渴病痹证”OR “消渴痹证”OR “周痹”）AND（“随机对照试验”OR “随机”OR “对照”OR “试验”OR “临床疗效观察”OR “疗效”OR “临床”OR “治疗”OR “影响”））**OR**  题名或关键词：（（“针刺”OR“针灸”OR“电针”OR “温针”OR “温针灸”OR “体针”OR “头针”OR “耳针”OR “穴位”）AND （“2型糖尿病”OR “非依赖胰岛素型糖尿病”OR “成人发病型糖尿病”OR “消渴”OR “消渴病”） AND （“糖尿病性周围神经病”OR “糖尿病周围神经病”OR “糖尿病周围神经病变”）AND（“2型糖尿病性周围神经病”OR “2型糖尿病性周围神经病变”OR “消渴病痹症”OR “消渴痹症”OR “消渴病痹证”OR “消渴痹证”OR “周痹”）AND（“随机对照试验”OR “随机”OR “对照”OR “试验”OR “临床疗效观察”OR “疗效”OR “临床”OR “治疗”OR “影响”））**OR**  摘要：（（“针刺”OR“针灸”OR“电针”OR “温针”OR “温针灸”OR “体针”OR “头针”OR “耳针”OR “穴位”）AND （“2型糖尿病”OR “非依赖胰岛素型糖尿病”OR “成人发病型糖尿病”OR “消渴”OR “消渴病”） AND （“糖尿病性周围神经病”OR “糖尿病周围神经病”OR “糖尿病周围神经病变”）AND（“2型糖尿病性周围神经病”OR “2型糖尿病性周围神经病变”OR “消渴病痹症”OR “消渴痹症”OR “消渴病痹证”OR “消渴痹证”OR “周痹”）AND（“随机对照试验”OR “随机”OR “对照”OR “试验”OR “临床疗效观察”OR “疗效”OR “临床”OR “治疗”OR “影响”））NOT ((主题：动物NOT人类+人)OR(题名或关键词：动物NOT人类+人)OR(摘要：动物NOT人类+人)) |

| Search strategy in VIP |
| --- |
| ((**M=**针刺+针灸+电针+温针+温针灸+体针+头针+耳针+穴位) AND (**M=** 2型糖尿病+非依赖胰岛素型糖尿病+成人发病型糖尿病+消渴+消渴病)AND (**M=** 糖尿病性周围神经病+糖尿病周围神经病+糖尿病周围神经病变)AND (**M=**2型糖尿病性周围神经病+2型糖尿病性周围神经病变+消渴病痹症+消渴痹症+消渴病痹证+消渴痹证+周痹)AND (**M=**随机对照试验+随机+对照+试验+临床疗效观察+疗效+临床+治疗+影响))**OR**  ((K**=**针刺+针灸+电针+温针+温针灸+体针+头针+耳针+穴位) AND (K**=** 2型糖尿病+非依赖胰岛素型糖尿病+成人发病型糖尿病+消渴+消渴病)AND (K**=** 糖尿病性周围神经病+糖尿病周围神经病+糖尿病周围神经病变)AND (K**=**2型糖尿病性周围神经病+2型糖尿病性周围神经病变+消渴病痹症+消渴痹症+消渴病痹证+消渴痹证+周痹)AND (K**=**随机对照试验+随机+对照+试验+临床疗效观察+疗效+临床+治疗+影响)) **OR**  ((**R=**针刺+针灸+电针+温针+温针灸+体针+头针+耳针+穴位) AND (**R=** 2型糖尿病+非依赖胰岛素型糖尿病+成人发病型糖尿病+消渴+消渴病)AND (**R=** 糖尿病性周围神经病+糖尿病周围神经病+糖尿病周围神经病变)AND (**R=**2型糖尿病性周围神经病+2型糖尿病性周围神经病变+消渴病痹症+消渴痹症+消渴病痹证+消渴痹证+周痹)AND (**R=**随机对照试验+随机+对照+试验+临床疗效观察+疗效+临床+治疗+影响)) NOT ((**M=**动物NOT人类+人)OR(K**=**动物NOT人类+人)OR(**R=**动物NOT人类+人)) |

| Search strategy in SinoMed |
| --- |
| （（"针刺"[常用字段:智能] OR "针灸"[常用字段:智能] OR "电针"[常用字段:智能] OR "温针"[常用字段:智能] OR "温针灸"[常用字段:智能] OR "体针"[常用字段:智能] OR "头针"[常用字段:智能] OR "耳针"[常用字段:智能] OR "穴位"[常用字段:智能]）AND （"2型糖尿病"[常用字段:智能] OR "非依赖胰岛素型糖尿病"[常用字段:智能] OR "成人发病型糖尿病"[常用字段:智能] OR "消渴"[常用字段:智能] OR "消渴病"[常用字段:智能]） AND （"糖尿病性周围神经病"[常用字段:智能] OR "糖尿病周围神经病"[常用字段:智能] OR "糖尿病周围神经病变"[常用字段:智能]）AND（"2型糖尿病性周围神经病"[常用字段:智能] OR "2型糖尿病性周围神经病变"[常用字段:智能] OR "消渴病痹症"[常用字段:智能] OR "消渴痹症"[常用字段:智能] OR "消渴病痹证"[常用字段:智能] OR "消渴痹证"[常用字段:智能] OR "周痹"[常用字段:智能]）AND（"随机对照试验"[常用字段:智能] OR "随机"[常用字段:智能] OR "对照"[常用字段:智能] OR "试验"[常用字段:智能] OR "临床疗效观察"[常用字段:智能] OR "疗效"[常用字段:智能] OR "临床"[常用字段:智能] OR "治疗"[常用字段:智能] OR "影响"[常用字段:智能]））**OR**  （（"针刺"[全部字段:智能] OR "针灸"[全部字段:智能] OR "电针"[全部字段:智能] OR "温针"[全部字段:智能] OR "温针灸"[全部字段:智能] OR "体针"[全部字段:智能] OR "头针"[全部字段:智能] OR "耳针"[全部字段:智能] OR "穴位"[全部字段:智能]）AND （"2型糖尿病"[全部字段:智能] OR "非依赖胰岛素型糖尿病"[全部字段:智能] OR "成人发病型糖尿病"[全部字段:智能] OR "消渴"[全部字段:智能] OR "消渴病"[全部字段:智能]） AND （"糖尿病性周围神经病"[全部字段:智能] OR "糖尿病周围神经病"[全部字段:智能] OR "糖尿病周围神经病变"[全部字段:智能]）AND（"2型糖尿病性周围神经病"[全部字段:智能] OR "2型糖尿病性周围神经病变"[全部字段:智能] OR "消渴病痹症"[全部字段:智能] OR "消渴痹症"[全部字段:智能] OR "消渴病痹证"[全部字段:智能] OR "消渴痹证"[全部字段:智能] OR "周痹"[全部字段:智能]）AND（"随机对照试验"[全部字段:智能] OR "随机"[全部字段:智能] OR "对照"[全部字段:智能] OR "试验"[全部字段:智能] OR "临床疗效观察"[全部字段:智能] OR "疗效"[全部字段:智能] OR "临床"[全部字段:智能] OR "治疗"[全部字段:智能] OR "影响"[全部字段:智能]））**OR**  （（"针刺"[核心字段:智能] OR "针灸"[核心字段:智能] OR "电针"[核心字段:智能] OR "温针"[核心字段:智能] OR "温针灸"[核心字段:智能] OR "体针"[核心字段:智能] OR "头针"[核心字段:智能] OR "耳针"[核心字段:智能] OR "穴位"[核心字段:智能]）AND （"2型糖尿病"[核心字段:智能] OR "非依赖胰岛素型糖尿病"[核心字段:智能] OR "成人发病型糖尿病"[核心字段:智能] OR "消渴"[核心字段:智能] OR "消渴病"[核心字段:智能]） AND （"糖尿病性周围神经病"[核心字段:智能] OR "糖尿病周围神经病"[核心字段:智能] OR "糖尿病周围神经病变"[核心字段:智能]）AND（"2型糖尿病性周围神经病"[核心字段:智能] OR "2型糖尿病性周围神经病变"[核心字段:智能] OR "消渴病痹症"[核心字段:智能] OR "消渴痹症"[核心字段:智能] OR "消渴病痹证"[核心字段:智能] OR "消渴痹证"[核心字段:智能] OR "周痹"[核心字段:智能]）AND（"随机对照试验"[核心字段:智能] OR "随机"[核心字段:智能] OR "对照"[核心字段:智能] OR "试验"[核心字段:智能] OR "临床疗效观察"[核心字段:智能] OR "疗效"[核心字段:智能] OR "临床"[核心字段:智能] OR "治疗"[核心字段:智能] OR "影响"[核心字段:智能]））**NOT** （（"动物"[常用字段:智能] NOT "人"[常用字段:智能] OR "人类"[常用字段:智能]）OR （"动物"[全部字段:智能] NOT "人"[全部字段:智能] OR "人类"[全部字段:智能]）OR（"动物"[核心字段:智能] NOT "人"[核心字段:智能] OR "人类"[核心字段:智能]）） |
